# Phosphate-Trapping Liposomes for Long-Term Management of Hyperphosphatemia

**DOI:** 10.3390/ma15217779

**Published:** 2022-11-04

**Authors:** Chen Tzror-Azankot, Adi Anaki, Tamar Sadan, Menachem Motiei, Rachela Popovtzer

**Affiliations:** Faculty of Engineering and the Institute of Nanotechnology & Advanced Materials, Bar-Ilan University, Ramat Gan 5290002, Israel

**Keywords:** phosphate binder, liposomes, hyperphosphatemia, ferric citrate

## Abstract

Hyperphosphatemia is a typical complication of end-stage renal disease, characterized by elevated and life-threatening serum phosphate levels. Hemodialysis does not enable sufficient clearance of phosphate, due to slow cell-to-plasma kinetics of phosphate ions; moreover, dietary restrictions and conventional treatment with oral phosphate binders have low success rates, together with adverse effects. Here, we developed a new concept of phosphate-trapping liposomes, to improve and prolong the control over serum phosphate levels. We designed liposomes modified with polyethylene glycol and encapsulated with the phosphate binder ferric citrate (FC liposomes). These liposomes were found to trap phosphate ions in their inner core, and thereby lower free phosphate ion concentrations in solution and in serum. The FC liposomes showed higher phosphate binding ability as phosphate concentrations increased. Moreover, these liposomes showed a time-dependent increase in uptake of phosphate, up to 25 h in serum. Thus, our findings demonstrate effective long-term phosphate trapping by FC liposomes, indicating their potential to reduce serum phosphate toxicity and improve current management of hyperphosphatemia.

## 1. Introduction

End-stage renal disease is an irreversible loss of kidney function, which necessitates a regular course of hemodialysis, ideally followed by kidney transplant, for patient survival [1]. A major complication of the disease is hyperphosphatemia, characterized by elevated phosphate levels in blood. This phenomenon leads to the development of calcium-phosphate deposits in blood vessels and other tissues, skeleton damage and increased risk of fractures, cardiovascular disease, and mortality [2,3,4,5,6,7]. Hence, control of hyperphosphatemia is essential for end-stage renal disease patients.

Hemodialysis alone does not enable sufficient clearance of phosphate from blood, as opposed to other small molecules. During a hemodialysis session, blood concentrations of small molecules (e.g., creatinine and urea) decrease as a function of time via the dynamic equilibrium between their intracellular and extracellular distribution. However, phosphate’s intradialytic kinetics are distinctly characterized by an initial rapid decrease in its blood concentration, which subsequently reach a plateau due to the ion’s slow shift from cells to plasma. This distribution can cause significant post-dialytic rebound of serum phosphate, i.e., rapid return to high, pre-hemodialysis serum phosphate levels [2,8,9,10,11]. Thus, to date, patients’ phosphate levels are managed by a combination of dietary phosphate restriction and orally administered phosphate binders [11,12,13]. However, dietary restriction requires significant lowering of protein intake, which can induce malnutrition associated with increased mortality [3,13]. Current phosphate binders have poor solubility and bioavailability and are therefore used orally to decrease the gastrointestinal absorption of phosphorus [12,13]. While these binders reduce phosphate absorption into blood, their efficacy is nonetheless suboptimal, and they generally cannot improve clinical outcome [14]. Moreover, treatment with phosphate binders entails a high-burden regimen of multiple daily pills [15] and can have serious adverse effects [14] leading to substantial non-compliance of patients, which further impairs management of serum phosphate levels [6,9,16].

Liposomes are spherical nano-vesicles formed by concentric lipid bilayers enclosing an aqueous core. Liposomes are emerging as efficient drug delivery systems due to their bioavailability, enhancement of drug solubility, and prolonged half-life in circulation [17,18,19,20]. The latter can be achieved by modification of liposomes with polyethylene glycol (PEG) chains; a known example is Doxil, a US Food and Drug Administration (FDA)-approved liposomal chemotherapy carrier with a circulation lifetime of 20–30 h [17,21]. Thus, encapsulating phosphate binders within PEGylated liposomes could serve as a new approach to improve control of serum phosphate levels. Liposomes have the potential to improve phosphate binder’s solubility, enable intravenous instead of oral administration, and provide prolonged action in circulation. In particular, the long circulation lifetime of liposomes can correspond with the continuous increase in serum phosphate levels after hemodialysis. Trapping serum phosphate within liposomes, in turn, can potentially promote further cellular release of phosphate for further clearance by liposomes.

**Figure 1:**
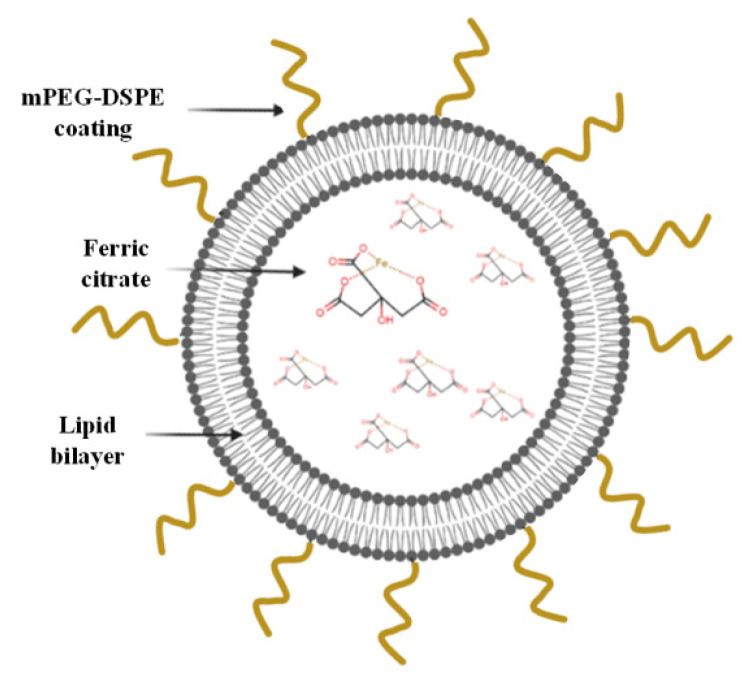
Schematic representation of the FC liposomes.

Here, PEGylated liposomes were developed based on the composition of long-circulating Doxil and encapsulating the phosphate binder ferric citrate (FC) (Figure 1). FC is a relatively new oral phosphate binder that can decrease serum phosphate levels in patients, yet it entails the same abovementioned challenges as other phosphate binders [7,12,22,23]. As initial proof of concept, in the present study we demonstrate that encapsulating FC within liposomes time-dependently uptakes phosphate from serum and decreases serum phosphate levels to a physiologically desired range, by trapping phosphate ions within the liposomes’ inner core.

## 2. Materials and Methods

### 2.1. Liposome Synthesis

PEGylated liposomes were synthesized based on the well-known filter-extrusion method [24]. Briefly, 382.1 mg of hydrogenated soybean phosphatidylcholine (HSPC, LIPOID, Newark, NJ, USA), 87 mg of cholesterol (Sigma-Aldrich, St. Louis, MO, USA), and 105.2 mg of 1,2-distearoyl-sn-glycero-3-phosphoethanolamine-N-[methoxy(polyethylene glycol)-2000] (18:0 PEG2000 PE, Avanti polar lipids, Alabaster, AL, USA) at molar ratios 65:30:5 were dissolved in 1 mL of absolute EtOH (Sigma-Aldrich) at 65 °C. Then, 100 mg of FC (Sigma-Aldrich) was dissolved in warm 9.5 mL of HEPES (4-(2-hydroxyethyl)-1-piperazineethanesulfonic acid)-buffered saline (10 mM HEPES, 150 mM sodium chloride (NaCl), pH = 7.4) to reach a final lipid concentration of 75 mM, and solution pH was adjusted to 6 as iron precipitates at pH > 7, while soluble FC complexes are formed at pH above 3.0 [25]. The organic phospholipid solution was mixed rapidly with aqueous solution at 65 °C, and the liposomes were downsized by stepwise extrusion, using LiposoFast-50 instrument (Avestin, Inc., Ottawa, ON, Canada) with polycarbonate membranes (Whatman, Maidstone, UK) up to 200 nm pore size. Excess FC was washed using overnight dialysis with HEPES-buffered saline (10 mM HEPES, 150 mM NaCl, pH = 7.4), followed by column separation using a prepacked PD-10 desalting column (GE Healthcare UK Ltd., Buckinghamshire, UK) to ensure soluble content. As control, liposomes without FC (empty liposomes) were also prepared using the same procedure but without FC dissolved in aqueous buffer.

Liposomes were characterized by ζ potential and dynamic light scattering (ZetaSizer 3000 HS, Malvern Instruments, Malvern, UK). For the cryo-EM measurement, 3 μL of liposome samples were loaded on a glow discharged (EmiTech K100 machine) lacey grid, blotted and plunged into liquid ethane using a Gatan CP3 automated plunger, and stored in liquid nitrogen until use. Frozen specimens (samples with liposomes embedded in vitreous ice) were transferred to Gatan 914 cryo-holder and maintained at temperatures below −176 °C inside the microscope. Samples were inspected with a Tecnai G2 microscope (FEI, Hillsboro, OR, USA) with an acceleration voltage of 120 kV, which is equipped with a cryobox decontaminator. Images were taken using a digital micrograph with a multi-scan camera model 794 (Gatan Inc., Pleasanton, CA, USA) in different resolutions. To ensure FC encapsulation, quantitative elemental analysis of iron was performed using inductively coupled plasma optical emission spectrometry (ICP-OES; Agilent Technologies, Santa Clara, CA, USA). Calibration curve with known iron concentrations (0, 0.01, 0.05, 0.1, 0.5, 1, and 5 mg/L) was prepared and iron concentration was determined according to absorbance values, compared with calibration curves. To ensure that FC does not leak from the liposomes over time, FC liposomes in solution were placed in a dialysis tube, and the presence of iron in the liposome solution, and in the dialysate, was measured by ICP-OES at several time points (6, 12, 24, and 48 h). The analysis sensitivity threshold was 0.2% leakage.

### 2.2. Incubation of Liposomes with Phosphate Solutions

To test the liposomes’ ability to trap free phosphate ions in solution, liposomes were incubated with phosphate solutions at increasing concentrations. To this end, phosphate solutions were prepared by a series of dilutions of sodium phosphate monohydrate (Sigma-Aldrich) dissolved in HEPES-buffered saline (10 mM HEPES, 150 mM NaCl, pH = 7.4) to yield solutions with increasing phosphate concentrations of 0.625, 1.25, 2.5, 5, 10, 20, 40, and 60 mg/dL. A dialysis tube with 1 mL FC liposomes (1.22 mg FC) was placed in 70 mL of each phosphate solution, or in HEPES with no phosphate, for 24 h at 37 °C under constant stirring. Elemental analysis of phosphorus in each solution before and after incubation with the liposomes was performed by ICP-OES. Phosphate binding efficiency was calculated by the following formula:

(1) $$\left(\left(\left[P\right]\;\left(t_{0}\right)\;\left(\frac{mg}{dL}\right)\right)-\left(\left[P\right]\;\left(t_{24}\right)\left(\frac{mg}{dL}\right)\right)\right)\cdot \frac{\;{\left[PO_{4}\right]}^{-3}Mw\;\left(\frac{gr}{mol}\right)}{\left[P\right]\;Mw\;\left(\frac{gr}{mo}\right)}={\left[PO_{4}\right]}^{-3}\;binding\left(\frac{mg}{ml\;FC\;LP}\right)$$

**Equation (1).** Calculation of phosphate binding efficiency (P: phosphorus, PO_4_^−3^: phosphate, t_24_: post 24 h of incubation with liposomes, FC LP: FC liposomes).

### 2.3. TLC Analysis

Liposomes with or without FC were incubated with phosphate solution (20 mg/dL) for 24 h, then free phosphate was removed from the solutions using a PD-10 desalting column. These liposomes, as well as FC liposomes without incubation with phosphate, were dissolved by adding 100 µL of liposome colloid solution to 500 µL of chloroform−methanol (2:1 *v*/*v*) solution, and 5 µL was loaded onto gel TLC glass plates. A chloroform−methanol−water (65:25:4 *v*/*v*/*v* (volumetric ratio)) solvent system was used for chromatographic separation. Plates were stained using ammonium molybdate. The loading spot was scraped from the TLC plate and placed in acid digestion overnight [26,27]. Phosphorus content was then quantified using ICP-OES to indicate the levels of phosphate in samples.

### 2.4. Analysis of Serum Treated with FC Liposomes

Serum was produced from defibrinated sheep blood (ThermoScientific, R54016, Milan, Italy) by centrifuging the blood at 2000× *g* for 10 min at 4 °C. The resulting supernatant was collected, apportioned into 0.5 mL aliquots, and stored at −20 °C until use. To measure the decrease in serum phosphate levels over time, serum (500 µL) was incubated with liposomes (100 µL, 75 mM, 0.122 mg FC) at 37 °C in a 5% humidified CO_2_ incubator for 0 (i.e., dialysis was performed directly after mixing), 2 h, 4 h, 12 h, and 24 h under constant stirring. As control, serum with 100 µL HEPES buffer was incubated at 37 °C (without liposomes) for the same time intervals. Following incubation, each solution was transferred into a dialysis tube and placed in 70 mL HEPES buffer for 24 h under constant stirring to allow unbound phosphate ions to diffuse into the buffer. Phosphate concentrations in the HEPES buffer were then quantified using ICP-OES.

To evaluate the ability of FC liposomes to lower phosphate levels in serum, we tested serum with different phosphate concentrations. Serum (500 µL), either its natural state or after addition of 5 µL or 10 µL of 2.5 mg/mL phosphate solution, was incubated with FC liposome solution (100 µL, 75 mM) for 24 h at 37 °C. Each serum sample (*n* = 3/group) was then transferred to dialysis tubes and placed in 70 mL HEPES buffer for 24 h under constant stirring, to allow unbound phosphate ions to diffuse into the buffer. Phosphate concentration in the HEPES buffer solution was measured using ICP-OES. Phosphate binding efficacy was calculated by the delta between the phosphorus concentration found in control serum (without liposomes) and in liposome-treated serum.

### 2.5. Statistical Analysis

Results are expressed as the mean ± SD. One-way analysis of variance (ANOVA) or repeated measures ANOVA and Tukey’s HSD post hoc tests were used to test the statistical significance of differences among groups for multiple comparisons. Statistical significance of the differences in the means of two groups was evaluated by using Student’s *t*-test. P values below 0.05 were considered statistically significant unless stated otherwise. Statistical analyses were performed in GraphPad Prism 7 (GraphPad Software, Inc., San Diego, CA, USA).

## 3. Results and Discussion

### 3.1. Synthesis and Characterization of FC Liposomes

**Figure 2:**
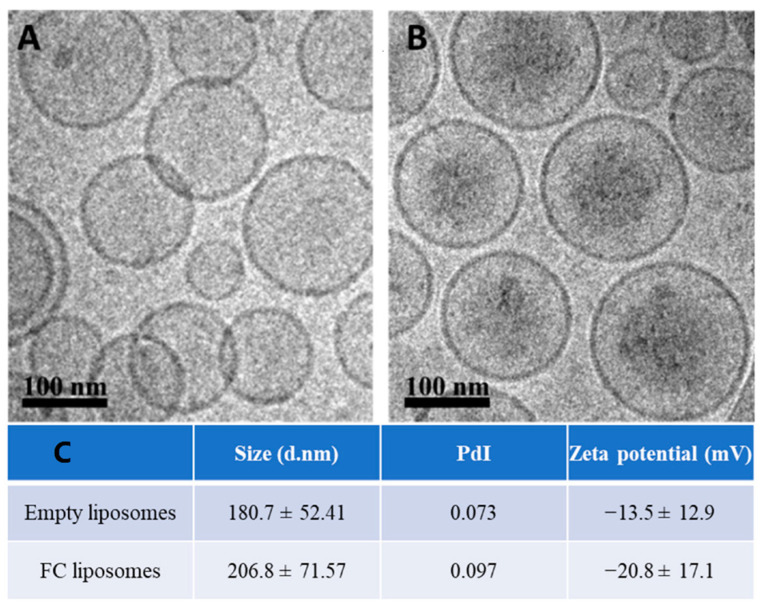
Characterization of liposomes encapsulating FC. Cryo−TEM images of (**A**) empty liposomes and (**B**) FC−liposomes (scale bar 100 nm). (**C**) Liposome size, polydispersity index (PdI), and zeta potential of empty liposomes and liposomes with FC.

Liposomes (200 nm) were synthesized using HSPC, cholesterol, and DSPE-mPEG (molar ratios of 65%, 30%, and 5%, respectively); FC was passively encapsulated in the liposomes’ inner core. The liposomes were characterized using cryogenic electron microscopy (Cryo-EM), zeta potential, and dynamic light scattering. Cryo-EM confirmed successful synthesis of the liposomes with FC in their inner core (Figure 2A,B). The liposomes showed an increase in their average size and a decrease in zeta potential due to FC loading, which further confirmed successful FC encapsulation (*p* > 0.05, Student’s *t*-test; Figure 2C). FC liposomes incubated in saline at room temperature for 15 days showed no significant changes in size and charge over time (Figure S1A). To measure FC encapsulation in the liposomes, inductively coupled plasma optical emission spectrometry (ICP-OES) was used for quantitative elemental analysis of iron, which confirmed the presence of Fe^3+^ in liposomal fractions (1.22 mg FC/1 mL of 75 mM liposomes). Moreover, ICP-OES analysis of iron concentrations in dialyzed FC liposome solution (Methods) confirmed that FC did not leak from the liposomes over time (up to 48 h; Figure S1B).

### 3.2. FC Liposomes Trap Free Phosphate Ions from Solution

**Figure 3:**
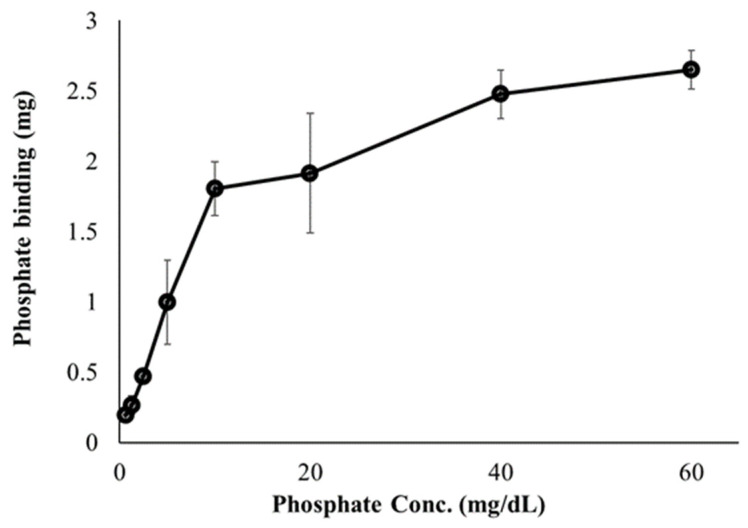
Effect of phosphate concentrations on phosphate binding by FC liposomes. FC liposomes (1 mL w/1.22 mg FC) were incubated with phosphate solutions at increasing concentrations for 24 h. Phosphate ion binding ability was evaluated by measuring phosphate concentrations in solutions before and after 24 h incubation with FC liposomes. Results presented as mean (of 3 samples) ± SD. Statistical significance was determined by one-way ANOVA with Tukey’s HSD test for multiple comparisons.

First, we investigated the ability of FC liposomes to trap free phosphate ions in buffer solutions. Dialysis tubes containing FC liposomes (1 mL, 75 mM) were incubated for 24 h in phosphate solutions at increasing concentrations (0, 0.625, 1.25, 2.5, 5, 10, 20, 40, and 60 mg/dL). Phosphorus content in the solutions (before and after incubation) were measured by ICP-OES, indicating levels of phosphate in the liposomes. We found decreased phosphate levels in the solutions following incubation with FC liposomes, indicating increased phosphate uptake into liposomes and its binding by encapsulated FC. Binding efficiency significantly increased as the concentration of phosphate in solution increased (one-way ANOVA followed by Tukey HSD post hoc, F = 65.46, *p* < 0.001; detailed in Supplementary Data), and stabilized at high phosphate concentrations (over 40 mg/dL), likely due to FC saturation (Figure 3).

Next, we verified that phosphate is indeed trapped within FC liposomes due to the phosphate binder FC, and not due to surface absorption. FC liposomes were incubated in phosphate solution (20 mg/dL) for 24 h, and free phosphate was then removed using a PD-10 desalting column. The FC liposomes were then dissolved and separated to their components using thin-layer chromatography (TLC), and elemental analysis of phosphorus in the liposomes’ water-soluble fraction was conducted by ICP-OES, indicating phosphate trapping. FC liposomes unexposed to phosphate solution and empty liposomes (without FC) exposed to phosphate solution served as controls.

**Figure 4:**
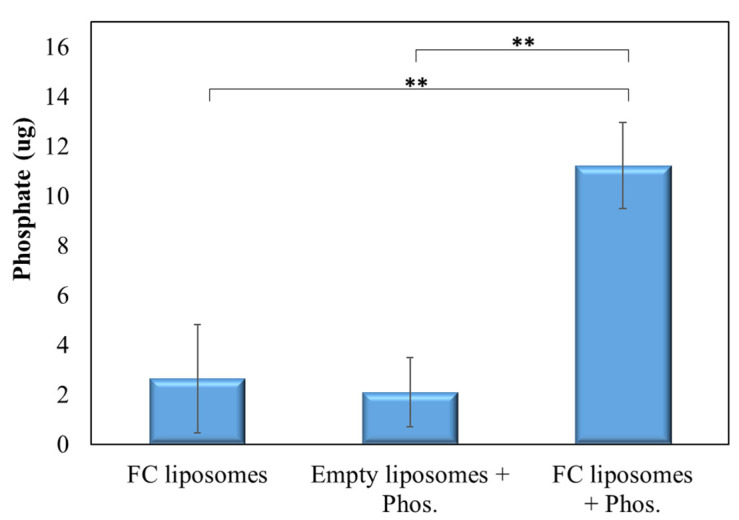
FC-mediated trapping of phosphate within liposomes. TLC analysis of phosphate presence in liposomes with or without encapsulated FC that were incubated with phosphate solution, or in non-incubated FC liposomes. Results show significantly higher phosphate presence in FC liposomes incubated with phosphate solution as compared to controls, confirming that phosphate was taken up into the liposomes’ water-soluble inner core and bound to FC. Results presented as mean (of 3 samples) ± SD. Statistical significance was determined by Student’s *t*-test; ** *p* < 0.01. Phos = phosphate.

Phosphate levels in pre-incubated FC liposomes were found to be significantly higher as compared to those in control non-incubated FC liposomes and in liposomes without FC (*p* < 0.01; Student’s *t*-test; Figure 4). These results confirm that phosphate was indeed trapped within the liposomes due to the presence of FC in their inner core.

### 3.3. FC Liposomes Reduce Serum Phosphate Levels

Next, the ability of the FC liposomes to lower phosphate levels in serum was evaluated. Sheep serum (500 µL), either without or with added phosphate (at 5 µL or 10 µL of 2.5 mg/mL), was incubated with FC liposomes (75 mM) for 24 h. Serum was then dialyzed for 24 h to allow diffusion of unbound phosphate ions to the dialysate, then measured by ICP-OES. Serum without liposomes served as control.

**Figure 5:**
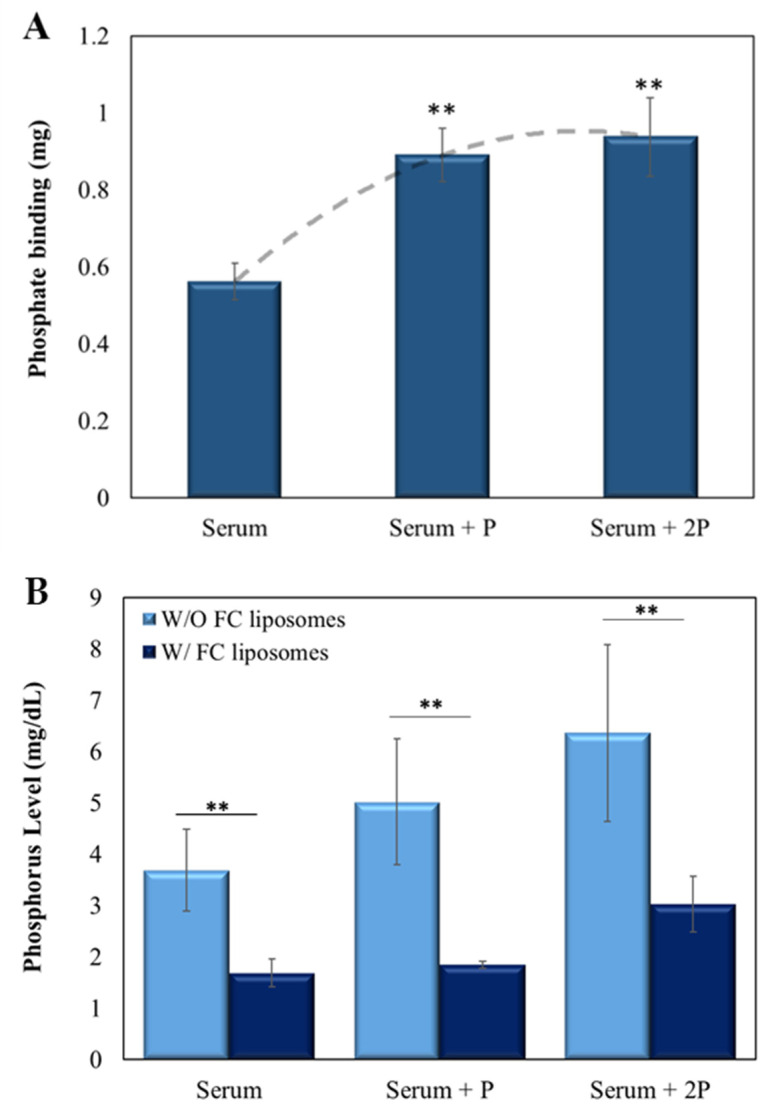
FC liposomes reduce phosphate levels in serum. Liposomes were incubated for 24 h with serum, either naive or with added phosphate (5 µL of 2.5 mg/mL phosphate (‘serum + P’) or 10 µL of 2.5 mg/mL phosphate (‘serum + 2P’)). (**A**) The amount of phosphate trapped in FC liposomes increased as serum phosphate concentrations increased. Binding reached saturation at high phosphate concentrations. Dotted line represents the binding trend of phosphate to FC liposomes; ** *p* < 0.01 for serum with added phosphate vs. serum w/o added phosphate (Student’s *t*-test). (**B**) Incubation of serum with FC liposomes resulted in a significant decrease in serum phosphorus levels (mg/dL) as compared to serum without (W/O) liposomes; ** *p* < 0.01, Student’s *t*-test. Results presented as mean (of 3 samples) ± SD.

FC liposomes were found to trap phosphate ions from serum, and the amount trapped increased with the increase in serum phosphate concentration (*p* < 0.01, serum with added phosphate vs. serum w/o added phosphate; Student’s *t*-test; Figure 5A). Similar binding abilities were found for the serum with added phosphate (5 µL and 10 µL of 2.5 mg/mL), likely due to FC saturation, which corresponds with the above finding in phosphate solution (shown in Figure 3). Moreover, a significant decrease in phosphorus concentrations in serum treated with FC liposomes was found as compared to those in untreated serum (*p* < 0.01, Student’s *t*-test; Figure 5B; values expressed in mg phosphorus/dL, in correspondence with clinically used values). Notably, a significant reduction in phosphorus concentrations was also seen in serum with either ~5 mg/dL or ~6 mg/dL phosphorus (Figure 5B), which are high concentrations found in hyperphosphatemia patients [28].

To ensure that complexing of phosphate to FC does not lead to acidification, we examined the pH of serum alone, serum immediately after addition of FC liposomes, and serum incubated with FC liposomes for 24 h. No detectable acidification of the two serum-FC liposome solutions was found as compared to the control serum.

### 3.4. FC Liposomes Uptake Phosphate from Serum over Time

**Figure 6:**
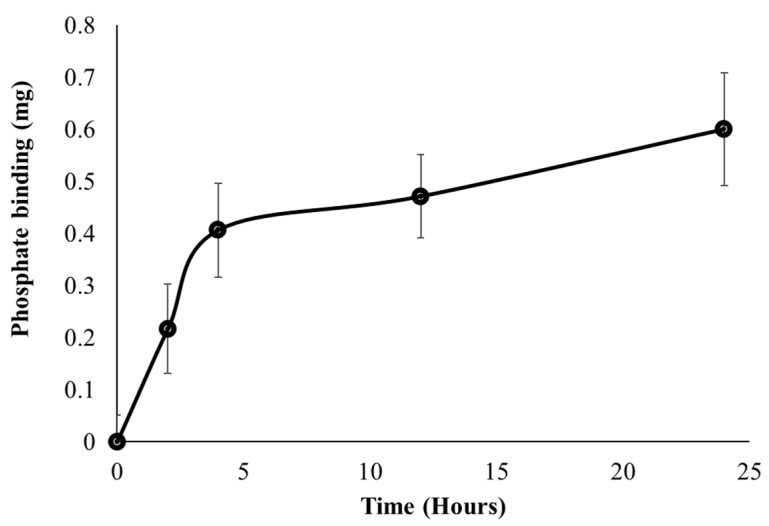
Uptake of phosphate from serum into liposomes over time. FC liposomes were incubated with serum, and phosphate uptake was evaluated at several intervals (0, 2, 4, 12, and 24 h). Phosphate binding ability is presented as the mean delta of phosphate levels in each sample before and after incubation with liposomes. Results presented as mean (of 3 samples) ± SD. Statistical significance was determined by repeated measures ANOVA with Tukey’s HSD test for multiple comparisons.

A major objective for developing the FC liposomes is long-term control of serum phosphate levels in circulation; therefore, we evaluated the FC liposome’s binding rate of phosphate over time. Serum was incubated with FC liposomes for increasing intervals of 0, 2, 4, 12, and 24 h. Serum was then dialyzed for 24 h, and ICP-OES was used to analyze phosphate concentrations in the dialysate, as compared to those in untreated serum (without liposome incubation). Phosphate uptake from serum into FC liposomes was found to increase over time. One-way repeated measures ANOVA test indicated a significant difference in the phosphate binding between the different time points (F = 31.77, *p* < 0.05). Tukey’s HSD post hoc test indicated significantly higher phosphate binding at 12 h as compared to 0 h, and at 24 h as compared to 0 h and 2 h. Phosphate uptake amounts were greater at early time points, up to 5 h, and then stabilized at later time points, as the liposomes approached their maximum phosphate binding capacity (Figure 6). These findings indicate that phosphate uptake from serum into the liposomes occurs via time-dependent diffusion.

## 4. Conclusions

In the current study, the phosphate binder FC was encapsulated within PEGylated liposomes and their ability to reduce serum phosphate levels was investigated. We found that the FC liposomes trapped phosphate ions in their inner core, and thereby lowered phosphate concentrations in artificial phosphate solutions and in serum. In both cases, the FC liposomes’ binding ability increased with the increase in phosphate concentrations. FC liposomes also showed long-term and increased phosphate binding capacity in serum, up to 25 h. The findings indicate that phosphate diffuses into the liposomes and binds to the phosphate binder, and thus enables significant reduction in serum phosphate levels—even at higher phosphorus concentrations of ~5 mg/dL and ~6 mg/dL, which are identical to the concentrations found in hyperphosphatemia patients [28].

PEGylated liposomes have unique biodistribution and pharmacokinetic profiles, resulting from their long circulation time and the stable retention of drugs in their water-soluble inner core. The pharmacokinetic profile of PEGylated liposomes can be clearly distinguished from those of the free drug and can therefore be used to optimize drug efficacy and reduce toxicity. Our results suggest that FC, which is currently given orally due to poor solubility and bioavailability, may be administered intravenously when encapsulated in liposomes. Additionally, as a complex of free FC and phosphate is not soluble in blood [29,30], encapsulation of FC in liposomes can improve the complex’s solubility and increase circulation time. Moreover, the finding that FC is not released from the liposomes suggests that FC liposomes may prevent the adverse effects caused by the free phosphate binder. Taken together, these unique characteristics indicate that FC liposomes have potential to serve as a phosphate trap in circulation, which can decrease serum phosphate concentrations, alleviate the burden involved in use of existing phosphate binders, and reduce their side effects.

Several recent studies have shown use of empty liposomes (with no encapsulated drug) to passively increase extraction of endogenous toxins, in order to augment dialysis in preclinical models [31]. These include liposomes with an acidic core for extracting ammonia in hepatic failure, or cationic, linoleic acid-based, or soy-phospholipid-based liposomes for improving removal of protein-bound endogenous toxins in renal failure. The current study is, to the best of our knowledge, the first to show that encapsulation of a phosphate binder within liposomes is effective for reducing serum phosphate levels—indicating their potential for improving management of hyperphosphatemia.

More studies are needed to establish whether the current findings can be implemented as an efficient and safe treatment for hyperphosphatemia. Various parameters need to be investigated, such as pharmacokinetics and clearance patterns. It is notable that due to the size (~200 nm) as well as the PEGylation of the FC liposomes, they are likely to avoid uptake by cells of the mononuclear phagocyte system and interact with hepatocytes in the liver, leading to clearance into the gastrointestinal tract and elimination by feces [32,33,34]. Future in vivo studies must be performed by creating a reliable animal model with elevated serum phosphate levels [35].

In conclusion, treatment with FC-encapsulating liposomes has potential to improve several parameters of dialysis treatment: it can minimize patient discomfort and enable controlled administration by injection immediately after dialysis sessions, which will also ensure treatment adherence (which is more challenging in the case of oral administration) by reducing the burden of existing medication regimens. FC liposomes have the potential to improve the clearance of phosphate during dialysis by prolonged and cumulative phosphate removal over time and present a promising approach for phosphate trapping to augment treatment of hyperphosphatemia.

## Data Availability

Not applicable.

## Supplementary Materials

The following supporting information can be downloaded at: https://www.mdpi.com/article/10.3390/ma15217779/s1, Figure S1: Characterization of liposomes encapsulating ferric citrate: (A) FC−liposomes size and zeta potential of liposomes with FC and phosphate. FC were incubated in saline at room temperature and their hydrodynamic size and zeta potential were measured over 15 days. The hydrodynamic size and zeta potential of FC−liposomes remained similar with no significant changes over the 15 days, indicating the good stability. (B) FC concentration in liposomes and the dialysate were measured over time, showing no leakage of FC from the liposomes. d = diameter. Results presented as mean (of 3 samples) +SEM.
