# Supplementary material for: Phosphate-Trapping Liposomes for Long-Term Management of Hyperphosphatemia
Source: Materials (Basel). 2022 Nov 4;15(21):7779. doi: 10.3390/ma15217779 (PMC9654424; doi:10.3390/ma15217779)
Supplement: Supplementary file 1 [file materials-15-07779-s001.zip › materials-1984623-supplementary.pdf]

*Supplementary Materials*

# Phosphate-Trapping Liposomes for Long-Term Management of Hyperphosphatemia

**Chen Tzror-Azankot, Adi Anaki, Tamar Sadan, Menachem Motiei and Rachela Popovtzer ***

Faculty of Engineering and the Institute of Nanotechnology & Advanced Materials, Bar-Ilan University, Ramat Gan 5290002, Israel
* Correspondence: rachela.popovtzer@biu.ac.il

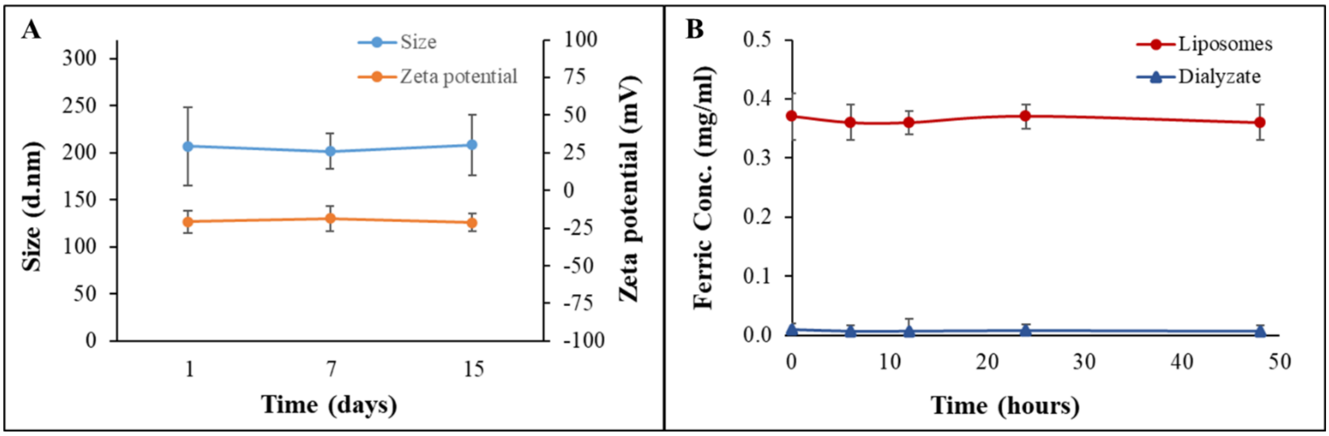


**Figure S1.** Characterization of liposomes encapsulating ferric citrate: (**A**) FC−liposomes size and zeta potential of liposomes with FC and phosphate. FC were incubated in saline at room temperature and their hydrodynamic size and zeta potential were measured over 15 days. The hydrodynamic size and zeta potential of FC−liposomes remained similar with no significant changes over the 15 days, indicating the good stability. (**B**) FC concentration in liposomes and the dialysate were measured over time, showing no leakage of FC from the liposomes. d = diameter. Results presented as mean (of 3 samples) ± SEM.

## *FC Liposomes Trap Free Phosphate Ions from Solution (figure 3)- Post-hoc Tukey HSD test*

Post-hoc Tukey HSD test for figure 3 indicated significant differences of the means as follows: Binding ability at phosphate concentration of 60 mg/dL was significantly higher than at 0.625, 1.25, 2,5, 5, 10, and 20 mg/dL. Binding ability at phosphate concentration of 40 mg/dL was significantly higher than at 0.625, 1.25, 2,5, 5, and 10 mg/dL. Binding ability at phosphate concentration of 20 mg/dL was significantly higher than at 0.625, 1.25, 2,5, and 5 mg/dL. Binding ability at phosphate concentration of 10 mg/dL was significantly higher than at 0.625, 1.25, 2,5, and 5 mg/dL. Binding ability at phosphate concentration of 5 mg/dL was significantly higher than at 0.625 and 1.25 mg/dL.
